# Does habitat disturbance affect stress, body condition and parasitism in two sympatric lemurs?

**DOI:** 10.1093/conphys/cow034

**Published:** 2016-09-10

**Authors:** Josué H Rakotoniaina, Peter M Kappeler, Pascaline Ravoniarimbinina, Eva Pechouskova, Anni M Hämäläinen, Juliane Grass, Clemens Kirschbaum, Cornelia Kraus

**Affiliations:** 1 Department of Sociobiology/Anthropology, Georg-August University of Göttingen, Kellnerweg 6, 37077 Göttingen, Germany; 2 Behavioral Ecology and Sociobiology Unit, Deutsches Primatenzentrum, Kellnerweg 4, 37077 Göttingen, Germany; 3 Helminthiasis Unit, Institut Pasteur of Madagascar, Ambatofotsikely, 101 Antananarivo, Madagascar; 4 Department of Biological Sciences, University of Alberta, Edmonton, Alberta, Canada T6G 2E9; 5 Department of Psychology, TU Dresden, Andreas-Schubert-Bau, Zellescher Weg 19, 01069 Dresden, Germany

**Keywords:** Body condition, habitat disturbance, lemurs, Madagascar, parasitism, stress

## Abstract

Understanding how animals react to human-induced changes in their environment is a key question in conservation biology. Owing to their potential correlation with fitness, several physiological parameters are commonly used to assess the effect of habitat disturbance on animals’ general health status. Here, we studied how two lemur species, the fat-tailed dwarf lemur (*Cheirogaleus medius*) and the grey mouse lemur (*Microcebus murinus*), respond to changing environmental conditions by comparing their stress levels (measured as hair cortisol concentration), parasitism and general body condition across four habitats ordered along a gradient of human disturbance at Kirindy Forest, Western Madagascar. These two species previously revealed contrasting responses to human disturbance; whereas *M. murinus* is known as a resilient species, *C. medius* is rarely encountered in highly disturbed habitats. However, neither hair cortisol concentrations nor parasitism patterns (prevalence, parasite species richness and rate of multiple infections) and body condition varied across the gradient of anthropogenic disturbance. Our results indicate that the effect of anthropogenic activities at Kirindy Forest is not reflected in the general health status of both species, which may have developed a range of behavioural adaptations to deal with suboptimal conditions. Nonetheless, a difference in relative density among sites suggests that the carrying capacity of disturbed habitat is lower, and both species respond differently to environmental changes, with *C. medius* being more negatively affected. Thus, even for behaviourally flexible species, extended habitat deterioration could hamper long-term viability of populations.

## Introduction

Habitat loss and degradation as a result of anthropogenic activities are major causes of species decline, and identifying their effect on the health and viability of wildlife populations is pivotal to conservation biology (Wikelski and Cooke, 2006; Acevedo-Whitehouse and Duffus, 2009). Anthropogenic disturbances can negatively affect individuals’ general health status; for instance, by altering resource availability, enhancing predation and hunting pressure or facilitating the spread of parasites (Keyser *et al*., 1998; Allan *et al*., 2003; Rode *et al*., 2006). However, despite this general trend, the sensitivity of an organism to changing environmental conditions is known to be highly species specific, and biological attributes, such as a slow life history and diet specialization, are good predictors of extinction risk (McKinney, 1997; Purvis *et al*., 2000; Stark *et al*., 2004; Cardillo *et al*., 2005).

Evaluating the physiological responses of animals to environmental changes can help to detect, monitor and—in the best case—prevent conservation problems (Cooke and O'Connor, 2010; Cooke *et al*., 2013). Yet, most studies that have focused on this aspect essentially investigated a single response variable, such as stress level or pattern of parasitism (Gillespie *et al*., 2005; Arroyo-Rodríguez and Dias, 2010; Rimbach *et al*., 2013). Despite the considerable utility of this approach for conservation biologists, it remains extremely arduous to predict how a given species will respond to disturbances or even to identify the intrinsic factors that can potentially initiate a population decline (Wasser *et al*., 1997; but see Creel *et al*., 2002). This difficulty is attributable to the fact that such a complex phenomenon is likely to involve multiple explanatory factors, as indicated by several long-term studies (Gulland, 1992; Milton, 1996), highlighting the need for using multiple indicators in health assessment studies.

Owing to their potential correlation with fitness, stress hormones [glucocorticoids (GCs), i.e. cortisol for most mammals], general body condition and parasite infection status are commonly used as health indicators and to assess the ability of wild populations to cope with environmental challenges (Wikelski *et al*., 2002; Chapman *et al*., 2007). Indeed, an increase in GC concentration is known to be adaptive when being exposed to an acute stressor (Sapolsky *et al*., 2000; Charmandari *et al*., 2005). In chronically stressful conditions, prolonged hypothalamic–pituitary–adrenal axis activity and elevated GC concentrations may induce various pathological effects and may eventually affect individual fitness negatively by reducing investment in reproduction and immune function (Romero, 2004; Dhabhar, 2009). Given that habitat degradation constitutes a permanent stressor for wild populations, studies that attempt to identify chronically stressed animals commonly assume GC concentrations to be higher in disturbed habitats than in undisturbed ones, although chronic stress could also lead to hypothalamic–pituitary–adrenal axis hypoactivity. The evidence so far is inconclusive, because increases, decreases or no changes in GC concentrations have been observed when comparing animals in disturbed and undisturbed habitats (reviewed by Dickens and Romero, 2013).

General body condition is commonly assumed to have an impact on animals’ fitness and health and has been defined as a gauge of an individual's energy reserves (Krebs and Singleton, 1993; Schulte-Hostedde *et al*., 2001). Maintenance and reproduction during periods of food scarcity are energetically challenging and therefore make metrics of body condition of primary interest for conservation biologists. Chronic stress consistently leads to a declining body mass, although the magnitude of this effect is species specific (reviewed by Dickens and Romero, 2013).

Infection with helminths and protozoa can also impair host fitness by affecting survival and/or reproduction. Parasites can severely alter the health of the host in various ways, ranging from tissue damage and blood loss to death or, more commonly, they interfere with basic functions; for example, by decreasing nutrient absorption or increasing energy expenditure (Behnke, 1990; Nunn and Altizer, 2006). Yet, the link between infectious disease risk and habitat disturbance remains unclear, and the ‘dilution effect’, the main hypothesis that was proposed to explain this relationship, lacks consistent empirical support (Young *et al*., 2013). Indeed, an increase of infection risk with both a decrease (‘dilution effect’) and an increase (‘amplification effect’) in biodiversity have been found in wild populations (Hechinger and Lafferty, 2005; Keesing *et al*., 2006; Jones *et al*., 2008).

Here, we investigated the effect of human disturbance on GC concentration, general body condition and patterns of parasitism in two small-bodied sympatric lemur species, the fat-tailed dwarf lemur (*Cheirogaleus medius*) and the grey mouse lemur (*Microcebus murinus*), in western Madagascar. These closely related nocturnal species exhibit contrasting life-history characteristics and apparently differ in their vulnerability to habitat disturbance. *Microcebus murinus* is a disturbance-tolerant species and can persist in every forest type, even in small forest fragments (Ganzhorn *et al*., 2003, 2013). During periods of food scarcity, *M. murinus* can adopt multiple energy-saving strategies, such as flexible daily torpor or hibernation, and is able to switch between both strategies (Schmid and Ganzhorn, 2009; Vuarin *et al*., 2013). Moreover, *M. murinus* has a relatively fast life history characterized by one or two litters per year (Schmelting *et al*., 2000), a short lactation length and sexual maturity after 10 months (Martin, 1972; Perret, 1982; Eberle and Kappeler, 2006) that might enhance the species’ resilience to unpredicted or long-term stressors.

Unlike the grey mouse lemur, *C. medius* has life-history attributes that render this species more sensitive to challenging environmental conditions, such as later sexual maturity (within 2 years of age) and lower frequency of breeding cycle (once per year), with frequent skipping of reproduction (Fietz *et al*., 2000; Müller and Thalmann, 2002). *Cheirogaleus medius* are absent in very small forest fragments (Ganzhorn *et al*., 2003) and occur at low densities in degraded habitat (Schäffler *et al*., 2015), probably because of their specific dietary and shelter quality (high-insulation-capacity tree holes) requirements as a strict hibernator (Fietz and Ganzhorn, 1999; Dausmann, 2013). Their slower pace of life and habitat requirements might exacerbate their vulnerability to altered resource availability. Despite both species being classified as ‘least concern’ (IUCN, 2015), the assessment of the effects of human activities on the health of these two species can help in detection and understanding of the proximate mechanisms causing this difference in response to challenging conditions.

In this comparative field study on the specific links between anthropogenic disturbance and several health components, we therefore aimed to determine how these two lemur species respond physiologically to different levels of habitat disturbance. We predicted that a decrease in habitat quality would be linked with an increase in GC concentrations and parasitism levels and a decrease in general body condition. This effect should be more pronounced in *C. medius* compared with *M. murinus*, for which low or even no effect of anthropogenic disturbance might be expected.

## Materials and methods

### Study sites and populations

The study was carried out in the forest concession of Kirindy/Centre National de Formation, d'Etudes et de Recherche en Environnement et Foresterie (CNFEREF), which is part of a dry deciduous forest complex in central western Madagascar (central Menabe region). Four sites, locally known as N5, CS7, Savanna (SV) and Kirindy Village (KV), were selected because of their varying relative levels of current human disturbance. N5 and CS7 belong to the core area of the forest concession and have been used as long-term study sites for research activities since 1993. Although N5 is exclusively used for research, CS7 is also regularly frequented by small groups of tourists, both day and night. The SV constitutes the eastern border between the core forest area and a natural savannah, rendering it subject to edge effects. It is occasionally subjected to uncontrolled incursion because the forest constitutes a potential source of food and firewood for the local population. The KV study area is a forest fragment located close to a village and crossed by a path that is used daily by locals because it connects neighbouring villages. This forest fragment is rarely used for research activities and was identified as a suitable site for this study after a survey in February 2012 that confirmed the presence of both study species. Kirindy Village is the site most subjected to human incursion owing to its use as a source of fire and construction wood. The characteristics of the four study sites are summarized in Table 1.

**Table 1:** Characteristics of the four study sites in Kirindy Forest

Site	Habitat characteristic	Human activities	Distance to the closest village (km)	Distance to the closest clear area (km)
N5	Forest core	Research	7.88	5.78
CS7	Forest core	Research, tourism	9.00	5.19
SV	Forest edge	Research, food and firewood gathering	8.97	3.88
KV	Forest fragment	(Research), food, fire and construction wood gathering	3.59	2.3

Three of the study sites (N5, CS7 and SV) are equipped with a grid system of small foot trails at variable intervals (N5 and CS7, 25 m × 25 m; and SV, 100 m × 25 m). All research activities in KV were conducted along a 1.3 km transect (between 20°4′29.40″S, 44°37′10.50″E and 20°5′10.01″S, 44°37′0.47″E) passing through this forest fragment.

### Habitat structure characterization: density of shelter and food trees

Besides the level of anthropogenic disturbance among study sites, the habitats have not been previously characterized in terms of habitat parameters critical to the study species. To determine whether the level of human disturbance translates into a gradient of habitat quality, we first evaluated the apparent resource availability at each site by estimating densities of trees used for food and shelter by the two species. Trees with a diameter at breast height of >10 cm can be considered as a potential refuge (hibernation tree) for *C. medius* and *M. murinus* (Schmid, 1998; Dausmann, 2013). Big trees are also particularly targeted for construction woods by the local population, and we witnessed frequent illegal logging activities in KV. The density of potential shelter trees among study sites was assessed with the point-centred quarter method (Ganzhorn *et al*., 2011). In N5, CS7 and SV, path intersections were used as centre points, and the distance of the nearest tree with diameter at breast height >10 cm from a centre point was measured in each of the four quarters formed by these intersections. In KV, centre points were selected every 25 m on either side of the path, and quarters were obtained in all four compass directions. The number of sampling points differed between sites depending on the sampling design and area size (N5 = 453, CS7 = 80, SV = 90 and KV = 106).

The density of food trees for both species was assessed by using plots (5 m × 5 m). Each centre point used in the estimation of big tree density was taken to be one corner of the food tree plot. Within each plot, the number of tree species known to be consumed by either species was recorded (see Supplementary Table S1). A list of plant species eaten by *C. medius* is provided by Fietz and Ganzhorn (1999), and the feeding ecology of *M. murinus* is described by Dammhahn and Kappeler (2008).

### Sample collection and analysis

Populations of *C. medius* and *M. murinus* have been monitored using long-term live capturing in N5, CS7 and SV (Fietz, 1999; Fietz and Ganzhorn, 1999; Eberle and Kappeler, 2002), and an identical protocol was established in KV for this study. In brief, Sherman live traps baited with banana were set at each of the centre points used for habitat characterization late in the afternoon and checked early in the following morning for three consecutive days. Captured animals were individually tagged with a subcutaneous transponder (Trovan EURO ID, Germany), sexed at first capture, and morphometric measurements and body mass were recorded at subsequent monthly captures. All samples used to assess health indicators were collected during four distinct capture sessions: between September and December in 2012 and 2013 (hereafter ‘dry season’), which is a transition period from the dry to the rainy season, and between January and May in 2013 and 2014 (hereafter ‘rainy season’), which includes a transition period from the rainy to the dry season. The September–December period is characterized by low resource availability and covers the mating season of both species. Therefore, it is presumably an energetically demanding period for *M. murinus* and *C. medius*, in contrast to the January–May period, and that could lead to a contrasting effect on the health of individuals between both seasons. For instance, the chosen periods were shown to be extremes in terms of body mass for our study species (Fietz and Ganzhorn, 1999; Hämäläinen *et al*., 2014). Details of the number of individuals captured and samples used for all analyses are given in the Supplementary material, Tables S2 and S3.

#### Hair sampling for cortisol analysis

We collected one hair sample from the dorsocaudal region per individual and season to avoid potential variation of hair cortisol concentration (HCC) from different body regions (Macbeth *et al*., 2010; Carlitz *et al*., 2015). We used a pet grooming clipper (Aesculap Isis GT 420) to cut hair as close as possible to the skin. Samples were then kept at ambient temperature in 2 ml screw-cap Sarstedt tubes until shipment to the laboratory. Washing and extraction for hair cortisol analysis was performed at the University of Dresden (Germany), using minor modification from the protocol described by Gao *et al* (2013). In brief, samples were washed twice in 3 ml isopropanol for 3 min and dried under a fume hood. For cortisol extraction, 7.5 mg of hair was incubated with 40 μl internal standard and 2.4 ml methanol for 18 h at room temperature in a glass vial. Afterwards, samples were spun in a centrifuge at 11 180 g for 3 min, and 1.6 ml of the clear supernatant was dried at 50°C under a constant stream of nitrogen and resuspended using 175 μl double-distilled water. Of the final product, 100 μl was used for determination of the cortisol concentration. This assessment was performed using a Shimadzu HPLC–tandem mass spectrometry system (Shimadzu, Canby, OR, USA) coupled to an ABSciex API 5000 Turbo-ion-spray triple quadrupole tandem mass spectrometer (AB Sciex, Foster City, CA, USA), with purification by online solid-phase extraction (Gao *et al*., 2013). In total, we collected and analysed 502 *M. murinus* (N5 = 236, CS7 = 125, SV = 104 and KV = 37) and 184 *C. medius* (N5 = 143, CS7 = 16, SV = 10 and KV = 15) hair samples obtained during the four field seasons.

Unlike other matrices traditionally used in assessment of GC concentration (plasma, faeces, urine or saliva), hair offers a unique opportunity to measure cortisol accumulated during a longer time window (Stalder and Kirschbaum, 2012). Hair grows slowly; hence, GC concentrations in hair integrate several aspects of hypothalamic–pituitary–adrenal axis activity, such as baseline values, the magnitude of the stress response and the duration of elevated GC concentrations, over a period of several weeks to months. Only free (i.e. biologically active) GC is thought to be incorporated into the hair shaft (Davenport *et al*., 2006). There is growing evidence from direct and indirect validation studies that HCC is a useful measure of long-term stress load (Stalder and Kirschbaum, 2012; Carlitz *et al*., 2014; Grass *et al*., 2015). Recent studies have also found that HCC shows high intra-individual consistency (Stalder *et al*., 2012). Even though the long-term nature, the minimally invasive sampling and the long stability of HCC (Webb *et al*., 2010; González-de-la-Vara *et al*., 2011) render this method especially suitable for the field of conservation physiology, only few studies have so far applied the technique in this context (Martin and Reale, 2008; Macbeth *et al*., 2010; Bechshøft *et al*., 2012; Brearley *et al*., 2012; Carlitz *et al*., 2016).

#### Assessment of body condition

Individual body condition was estimated by using the scaled mass index (SMI), which reflects internal energy reserves of animals by taking into account the scaling relationship between body mass (BM) and a distinct measurement of body size (Peig and Green, 2009). This index therefore yields an individual value of body mass after standardizing it to the mean body size of all individuals present in the population. As recommended by Peig and Green (2009), head width (HW, the bizygomatic breadth), which is the body size measurement that has the highest correlation with body mass for our study species, was used as a size measurement (see also Vuarin *et al*., 2013). The scaled mass index for every individual *i* was calculated using the following formula:

$${\mathrm{SMI}}_{i}\;=\;M_{i}{\left({\mathrm{HW}}_{0}/{\mathrm{HW}}_{i}\right)}^{\mathrm{bSMA}}$$

where HW_0_ is the arithmetic mean of HW for our study population (*M. murinus* = 21 mm and *C. medius* = 26 mm). The scaling exponent bSMA (*M. murinus* = 4.976 and *C. medius* = 4.997), which is the slope of the standardized major axis regression of BM to HW, was obtained using the software RMA (Bohonak and Van der Linde, 2004). The SMI was calculated for 845 *M. murinus* (N5 = 534, CS7 = 159, SV = 116 and KV = 36) and 166 *C. medius* (N5 = 127, CS7 = 14, SV = 11 and KV = 14) individuals.

#### Faecal sampling for parasitology

Faecal samples were collected opportunistically from handling bags or traps and directly homogenized and stored in 2 ml screw-cap Sarstedt tubes with 10% formaldehyde after being weighed. Subsequent parasite analysis was conducted at the laboratories of the Deutsches Primatenzentrum (Germany) and the Institut Pasteur (Madagascar) using a slightly modified Ritchie's ether sedimentation method (Ritchie, 1948). Parasite eggs and oocysts were later retrieved from microscopic examination of faecal smears, and their shape, size and internal structure were used for parasite identification up to genus level (Raharivololona, 2006, 2009; Irwin and Raharison, 2009).

To describe the pattern of parasitism, we considered prevalence, parasite morphotype richness and the proportion of a population showing multiple infections (infected by more than one species of parasite), which have been linked to mortality and morbidity (Behnke, 1990; Raso *et al*., 2004). Parasite prevalence was calculated as the percentage of infected individuals among all examined animals from a population. To identify factors that can influence prevalence and multiple-species infection, we further considered them as response variables in models and coded as 1 if a given parasite morphotype was present (or if more than one species was recorded for multiple infection) and 0 if not. Morphotype richness was defined as the number of egg/oocyst definite types recorded in one individual and could indicate higher morbidity if it increases (Chapman *et al*., 2005). For a successful application of the method on *M. murinus*, see Hämäläinen *et al*. (2015b). During the field study period, 1167 *M. murinus* (N5 = 786, CS7 = 202, SV = 148 and KV = 31) and 186 *C. medius* (N5 = 145, CS7 = 16, SV = 13 and KV = 12) faecal samples were collected and analysed.

To control for potential observer bias, we used blind observation by coding samples before laboratory analysis of hair cortisol concentrations and faecal parasites.

### Statistical analysis

#### Habitat structure

Site differences in potential shelter density and food tree density were assessed with Kruskal–Wallis ANOVA, followed by pairwise *post hoc* Mann–Whitney *U*-tests. To account for multiple testing, we used false-discovery rate correction (Benjamini and Hochberg, 1995) at a threshold level *q* = 0.05. The statistical significance threshold was set at *P* < 0.05.

#### Health indicators

To examine between-site variation in HCC, we used a linear mixed model (LMM; Baayen, 2008) with site identity as a fixed factor for each species. Given the highly seasonal activity pattern of both species (Schmid and Kappeler, 1998; Dausmann *et al*., 2004, 2005), we included the fixed factor season in all subsequent models. Additionally, the factor sex was also taken into account, and as both sexes are subject to different pressures in different seasons because of their reproductive schedules, we accounted for the potential interaction between season and sex. This effect is expected to be more pronounced in *M. murinus*, where males terminate their torpor period before females (Schmid and Kappeler, 1998; Schmid, 1999) and might therefore experience higher stress during the dry season, whereas females might be energetically more stressed during gestation and weaning periods in the rainy season. Finally, age (juvenile, i.e. <1 year or adult) and its interaction with sex were added as fixed factors because the sensitivities of juveniles and adults to stressful conditions are known to differ significantly for many non-human primate species (Fourie and Bernstein, 2011; Hämäläinen *et al*., 2015a). Given that we obtained multiple samples from many individuals, identity was used as a random factor. Thus, the general model included the fixed factors site, season, sex and age and the interaction terms season × sex and age × sex. Logarithmic transformation was applied to HCC prior analyses to improve model fit.

The effect of anthropogenic disturbance on body condition was determined by computing a LMM on the log-transformed SMI. Like previous models, the general model included the fixed factors site, sex and age and the interaction terms season × sex and age × sex. For both HCC and SMI models, error variance homogeneity and normality were assessed using visual examination of residual plots of the full models.

The site difference in parasite prevalence and multiple-species infection were assessed using a binomial generalized linear mixed model with a logit link function for each study species. The factors season, sex and age and the interaction terms season × sex and age × sex were also included as fixed terms. Additionally, we controlled for the potential effect of faecal sample mass on the probability of finding gastrointestinal parasites. Sample mass was logarithmically transformed, centred and scaled. We could estimate predictors of prevalence for only the four most common helminths (*Hymenolepis*, *Subulura*, *Trichuris* and *Ascaris*) owing to the very low infection rate with other morphotypes. However, every distinct morphotype was taken into account when assessing morphotype richness. Furthermore, because of the low recapture rate of *C. medius* during rainy seasons, we could use data collected only during dry seasons for the fat-tailed dwarf lemur. Only dry season data were also used (factor season excluded) for the assessment of the pattern of infection with *Trichuris* in *M. murinus* owing to its low prevalence during the rainy season, raising issues of non-convergence and complete separation in the model. The low infection rate with *Trichuris* and *Ascaris* did not allow us to assess the effect of the interaction term sex × age when modelling the determinants of *Trichuris* prevalence in both species and *Ascaris* prevalence in *C. medius*.

Morphotype richness was analysed using a Poisson generalized linear mixed model with a log-link function and using the same fixed factors as with prevalence. Data from only the dry season were again used for *C. medius* because of the low recapture rate of *C. medius* during rainy seasons. General models of parasite morphotype richness were neither overdispersed (assessed using Pearson residuals; overdispersion parameter ɸ: *M. murinus* = 1.059 and *C. medius* = 0.866) nor zero-inflated (assessed using frequency plots).

Generally, to obtain better estimates of the fixed parameters, non-significant interaction terms and factors (*P* > 0.1) except for site were removed successively from the full models, and model comparisons were performed using likelihood ratio tests. Parameter estimates for factors that had a significant effect on health indicators are therefore reported from the reduced model. For all models, a random intercept structure was preferred over a random intercept and slope structure after a model selection based on AIC values. Models were fitted in R (version 3.2.2; R Core Team, 2015) with the lme4 R-package (Bates *et al*., 2015), and *P*-values were estimated using the Satterthwaite approximation implemented in the lmerTest package (Kuznetsova *et al*., 2015). Multiple comparisons between sites were performed with the R add-on package multcomp (Hothorn *et al*., 2008). The statistical significance threshold was set at *P* < 0.05. The reduced models for all analyses are given below, and the full models are reported in the Supplementary material (Tables S4, S5 and S6).

## Results

### Habitat structure

**Figure 1:**
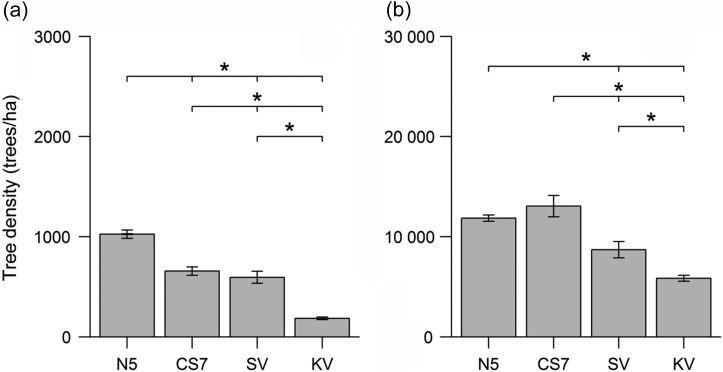
Density of potential shelter trees (**a**) and food trees (**b**) of *Microcebus murinus* and *Cheirogaleus medius* at the four study sites in Kirindy Forest. *P < 0.05.

Characterization of the habitat structure revealed that the gradient of anthropogenic disturbance between the four study sites was reflected in the density of potential shelter trees and partly observed in the density of food trees for *M. murinus* and *C. medius*. Indeed, the density of trees that could be used as a refuge (diameter at breast height >10 cm) differed significantly among the different sites (Kruskal–Wallis ANOVA: χ^2^_(3)_ = 235.69, *P* < 0.001; for all pairwise Mann–Whitney *U*-tests, *P* < 0.05), with a mean density of 1027 trees/ha for N5, 658 trees/ha for CS7, 596 trees/ha for SV and 186 trees/ha for KV (Fig. 1a). Moreover, although no difference was detected between N5 and CS7, significantly higher overall densities of food trees used by both species were found in N5 and CS7 compared with SV and KV but also in SV compared with KV (Kruskal–Wallis ANOVA: χ^2^_(3)_ = 118.42, *P* < 0.001; N5 vs. CS7, *P* = 0.77; and for all other pairwise Mann–Whitney *U*-tests, *P* < 0.05), with a mean of 11 858 trees/ha (N5), 13 060 trees/ha (CS7), 8716 trees/ha (SV) and 5864 trees/ha (KV; Fig. 1b). However, a large variation in the abundance of each tree species among the sites existed (Supplementary material, Table S1). Based on these data, N5 can be considered as the most suitable habitat for *M. murinus* and *C. medius*.

### Hair cortisol concentration

**Figure 2:**
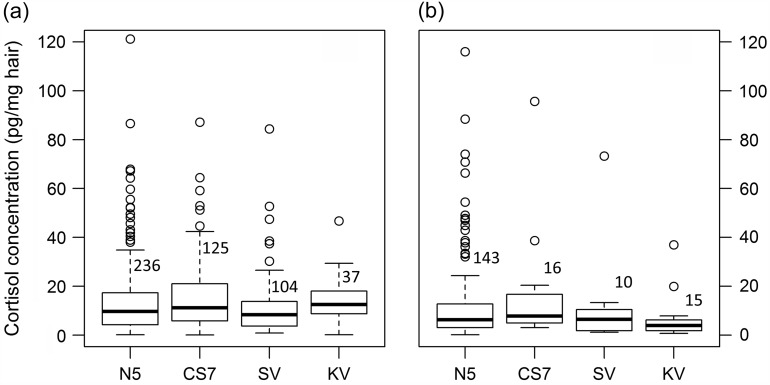
Variation in hair cortisol concentrations in *M. murinus* (**a**) and *C. medius* (**b**) among the four study sites. Numbers indicate the total number of hair samples analysed for each site.

As expected, the level of human disturbance had no effect on HCCs in *M. murinus* (LMM, χ^2^_(3)_ = 6.95, *P* = 0.07; Fig. 2a). Contrary to our predictions, the difference in habitat conditions caused by anthropogenic activities was not reflected by HCCs in *C. medius* either (LMM, χ^2^_(3)_ = 6.58, *P* = 0.11; Fig. 2b). Seasonal variation of HCC in both *M. murinus* and *C. medius* corresponded to our predictions based on their natural history. Significantly higher HCC values coupled with higher variation was observed in *C. medius* for the rainy season compared with the dry season (Table 2). For *M. murinus*, we found a sex difference in HCC, which seemed to be influenced by season (significant interaction term sex × season); males had higher average HCCs than females in the dry season, but the trend was reversed in the rainy season. Furthermore, we found higher average HCCs in juvenile mouse lemurs compared with adults (Table 2). Neither sex nor age had a significant effect on mean HCCs in *C. medius* (see Supplementary material, Table S4).

**Table 2:** Parameter estimates from reduced linear mixed models assessing variations of log (hair cortisol concentration) and log [general body condition (calculated as scaled mass index)] in *M. murinus* and *C. medius*

	*Microcebus murinus*				*Cheirogaleus medius*			
	Estimate	SE	*t*	*P*-value	Estimate	SE	*t*	*P*-value
Hair cortisol concentration								
Intercept	2.263	0.115	19.643	**<0.001**	1.850	0.155	11.897	**<0.001**
Site (ref. N5) CS7	0.250	0.113	2.202	**0.028**	0.293	0.265	1.105	0.271
SV	−0.344	0.123	−0.279	0.780	0.073	0.325	0.223	0.823
KV	0.224	0.181	1.238	0.216	−0.345	0.272	−1.269	0.206
Season (ref. dry)	0.265	0.138	1.922	0.055	1.260	0.188	6.707	**<0.001**
Sex (ref. female)	0.325	0.123	2.643	**0.008**				
Age (ref. juvenile)	−0.623	0.095	−6.571	**<0.001**	−0.314	0.167	−1.884	0.061
Sex × season	−0.561	0.185	−3.031	**0.002**				
Body condition (scaled mass index)								
Intercept	4.041	0.015	268.058	**<0.001**	4.810	0.016	304.126	**<0.001**
Site (ref. N5) CS7	0.006	0.014	0.447	0.728	−0.076	0.045	−1.685	0.094
SV	−0.022	0.016	−1.368	0.230	−0.001	0.050	−0.035	0.972
KV	0.131	0.027	4.807	**<0.001**	−0.017	0.045	−0.383	0.702
Season (ref. dry)	0.091	0.016	5.704	**<0.001**	0.233	0.031	7.591	**<0.001**
Sex (ref. female)	0.056	0.018	3.005	**0.002**				
Age (ref. juvenile)	0.051	0.017	3.032	**0.002**				
Sex × season	−0.101	0.022	−4.622	**<0.001**				
Sex × age	−0.043	0.022	−1.929	0.054				

Bold values indicate statistically significant results at the significance threshold *P* < 0.05.

### Body condition

**Figure 3:**
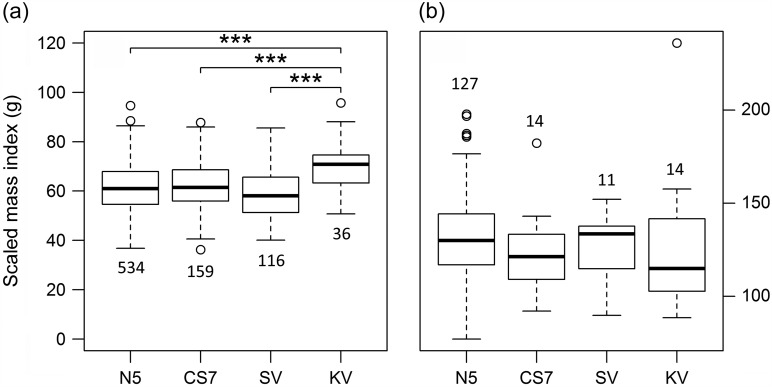
General body condition (measured as scaled mass index) of *M. murinus* and (**a**) *C. medius* (**b**) across the four study sites. ***P < 0.001. Numbers indicate the sample size for each site.

We found no significant difference in body condition among study sites in fat-tailed dwarf lemurs (LMM, χ^2^_(3)_ = 1.99, *P* = 0.57; Fig. 3b). However, mouse lemurs at KV showed significantly higher average SMI values than at the other sites (N5 vs. KV, *P* < 0.001; CS7 vs. KV, *P* < 0.001; SV vs. KV, *P* < 0.001; Fig. 3a and Table 2). Not surprisingly, this indicator of internal energy reserves was greater during the rainy season for both species, but this difference was more pronounced in *C. medius*. Again, a sex difference in body mass following a seasonal pattern (higher values for males in the dry season but reverse trend in the rainy season) was detected for *M. murinus*. Furthermore, adult mouse lemurs had a significantly higher body condition than juveniles (Table 2).

### Parasitism

In total, we identified 11 distinct egg morphotypes, with two of them (*Oesophagostomum* spp. and *Capillaria* spp.) found only in *M. murinus* (Table 3). Infection with multiple species was rather common in *M. murinus* and *C. medius*, and we detected a maximum of six distinct egg morphotypes in a single individual of both species. The occurrence of multiple-species infection (LMM, χ^2^_(3)_ = 6.70, *P* = 0.08; Table 4) and morphotype richness (LMM, χ^2^_(3)_ = 4.49, *P* = 0.21; Table 4) did not differ among sites for *M. murinus*. None of the components used to assess parasitism showed significant variation between sites for *C. medius*, but an age-specific sex difference in parasite morphotype richness was observed in *C. medius* (higher values for adult males; Table 4).

**Table 3:** Gastrointestinal parasites of *M. murinus* and *C. medius* in four different sites within Kirindy forest

	*Microcebus murinus*				*Cheirogaleus medius*			
	N5	CS7	SV	KV	N5	CS7	SV	KV
Total number of individuals	305	95	80	23	98	14	11	11
Multiple infections (%)	47.9	52.6	41.2	39.1	32.6	21.4	18.2	18.2
Prevalence (%) of Cestoda								
* Hymenolepis*	57	49.5	40	43.5	35.7	0	18.1	27.2
Nematoda								
*Subulura*	49.5	57.9	62.5	43.5	32.6	21.4	36.4	27.3
*Trichuris*	21.3	23.1	18.7	13	13.3	14.3	18.1	0
*Ascaris*	9.5	17.9	13.7	26.1	8.1	14.3	0	18.2
*Oxyuridae*	6.2	9.5	5	4.3	5.1	7.1	9.1	9.1
*Lemuricola*	1.6	0	1.2	0	1	0	0	9.1
*Oesophagostomum*	0	1	1.2	0				
*Capillaria*	0.6	0	0	0				
*Strongylida*	22.3	15.8	18.7	4.3	7.1	7.1	0	0
Trematoda								
*Opisthorchis*	0	1	0	0	1	0	0	0
*Metagonimus*	2.9	2.1	3.7	0	3.1	0	0	0

**Table 4:** Parameter estimates from reduced generalized linear mixed models assessing variation in multiple-morphotype infection rate and parasite morphotype richness in *M. murinus* and *C. medius*

	*Microcebus murinus*				*Cheirogaleus medius*			
	Estimate	SE	*z*	*P*-value	Estimate	SE	*z*	*P*-value
Multiple-morphotype infection								
Intercept	−1.478	0.178	−8.295	**<0.001**	−1.000	0.207	−4.838	**<0.001**
Site (ref. N5) CS7	0.463	0.194	2.391	**0.017**	−0.204	0.690	−0.295	0.768
SV	0.019	0.225	0.084	0.933	−0.705	0.796	−0.885	0.376
KV	0.444	0.430	1.031	0.302	−0.504	0.809	−0.623	0.533
Season (ref. dry)	−0.165	0.228	−0.726	0.468				
Sex (ref. female)	0.801	0.172	4.655	**<0.001**				
Age (ref. juvenile)	0.289	0.151	1.914	0.056				
Sex × season	−0.927	0.334	−2.772	**0.005**				
Parasite morphotype richness								
Intercept	−0.247	0.077	−3.220	**0.001**	0.039	0.282	0.140	0.888
Site (ref. N5) CS7	0.154	0.086	1.792	0.073	−0.223	0.403	−0.554	0.579
SV	0.035	0.098	0.355	0.723	−0.419	0.403	−1.038	0.299
KV	0.167	0.186	0.896	0.370	−0.295	0.415	−0.709	0.478
Season (ref. dry)	−0.185	0.101	−1.826	0.068				
Sex (ref. female)	0.354	0.075	4.728	**<0.001**	−0.557	0.534	−1.080	0.280
Age (ref. juvenile)	0.182	0.065	2.786	**0.005**	−0.517	0.329	−1.578	0.115
Sex × season	−0.321	0.145	−2.203	**0.028**				
Sex × age					1.188	0.588	2.020	**0.043**

Bold values indicate statistically significant results at the significance threshold *P* < 0.05.

The overall parasite prevalence (defined regardless of the helminth morphotype recorded) of the grey mouse lemur did not differ significantly among study sites (Table 5). However, among-site variation was observed for three out of the four most common morphotypes. In fact, the prevalence of *Hymenolepis* spp. eggs was significantly lower in SV compared with N5 (*z* = −2.99, *P* = 0.01), while *Subulura* spp. eggs were more frequently found in CS7 and SV compared with N5 (N5 vs. CS7, *z* = 4.03, *P* < 0.001; N5 vs. SV, *z* = 3.31, *P* = 0.004), and *Ascaris* spp. eggs had a significantly higher prevalence in the CS7 and KV populations compared with the N5 population (N5 vs. CS7, *z* = 2.72, *P* = 0.03; N5 vs. KV, *z* = 3.70, *P* = 0.001; Table 5). Moreover, *Hymenolepis* and *Trichuris* eggs had a higher prevalence in adult mouse lemurs, and sex and/or seasonal differences in prevalence were noticed for *Hymenolepis*, *Subulura*, *Trichuris* and *Ascaris* eggs (Table 5). Furthermore, the season-specific sex differences exhibited by *M. murinus* (higher values for males in the dry season but opposite trend in the rainy season) in other health indicators (HCC and SMI) were also observed for overall prevalence, morphotype richness and rate of multiple infections (Tables 4 and 5).

**Table 5:** Parameter estimates from reduced generalized linear mixed models assessing variation of parasite prevalence in *M. murinus* and *C. medius*

	*Microcebus murinus*				*Cheirogaleus medius*			
	Estimate	SE	*z*	*P*-value	Estimate	SE	*z*	*P*-value
Overall prevalence								
Intercept	0.038	0.155	0.245	0.806	0.009	0.198	0.483	0.629
Site (ref. N5) CS7	0.272	0.195	1.393	0.164	−0.251	0.618	−0.407	0.684
SV	0.102	0.218	0.467	0.640	−0.260	0.624	−0.418	0.676
KV	−0.032	0.420	−0.076	0.939	−0.272	0.667	−0.408	0.683
Season (ref. dry)	−0.288	0.201	−1.435	0.151				
Sex (ref. female)	0.756	0.169	4.482	**<0.001**				
Age (ref. juvenile)	0.339	0.142	2.388	**0.017**				
Sex × season	−0.635	0.294	−2.163	**0.031**				
*Hymenolepis*								
Intercept	−0.715	0.141	−5.066	**<0.001**	−0.916	0.239	−3.828	**<0.001**
Site (ref. N5) CS7	−0.042	0.170	−0.249	0.803	−3.618	2.163	−1.673	0.094
SV	−0.622	0.207	−2.999	**0.003**	−0.855	0.834	−1.025	0.305
KV	0.025	0.387	0.064	0.949	−0.701	0.850	−0.824	0.410
Season (ref. dry)	−0.295	0.145	−2.029	**0.042**				
Sex (ref. female)	0.232	0.129	1.802	0.071				
Age (ref. juvenile)	0.336	0.133	2.525	**0.011**				
*Subulura*								
Intercept	−1.205	0.143	−8.450	**<0.001**	−1.000	0.207	−4.838	**<0.001**
Site (ref. N5) CS7	0.767	0.190	4.035	**<0.001**	−0.204	0.690	−0.295	0.768
SV	0.715	0.215	3.319	**<0.001**	0.189	0.635	0.298	0.766
KV	0.458	0.425	1.079	0.280	0.019	0.708	0.227	0.978
Season (ref. dry)	−0.096	0.219	−0.440	0.660				
Sex (ref. female)	0.926	0.168	5.501	**<0.001**				
Sex × season	−1.193	0.325	−3.665	**<0.001**				
*Trichuris*								
Intercept	−3.139	0.384	−8.173	**<0.001**	−2.954	1.858	−1.589	0.112
Site (ref. N5) CS7	0.071	0.375	0.190	0.850	0.267	1.101	0.243	0.808
SV	−0.562	0.417	−1.347	0.178	0.645	1.184	0.545	0.586
KV	0.182	0.822	0.222	0.825	−2.423	2.539	−0.854	0.340
Sex (ref. female)	1.016	0.295	3.440	**<0.001**				
Age (ref. juvenile)	0.538	0.268	2.006	**0.045**				
*Ascaris*								
Intercept	−3.031	0.194	−15.585	**<0.001**	−10.026	1.568	−6.393	**<0.001**
Site (ref. N5) CS7	0.929	0.319	2.913	**0.003**	0.333	2.639	0.126	0.900
SV	0.597	0.366	1.635	0.102	−0.893	3.392	−0.263	0.792
KV	1.952	0.504	3.877	**<0.001**	0.480	2.703	0.178	0.859
Season (ref. dry)	−1.174	0.412	−2.852	**0.004**				
Sample mass	−0.225	0.127	−1.775	0.076				

Bold values indicate statistically significant results at the significance threshold *P* < 0.05.

## Discussion

In this study, we assessed the effect of anthropogenic disturbance on the health of two species that differ in their ability to cope with changing conditions and therefore added to the growing body of research focusing on the impact of human disturbance on lemur health (Irwin, 2008a; Bublitz *et al*., 2015). Concordant with its status as a disturbance-resistant species, *M. murinus* did not show signs of health deterioration in habitats subjected to greater human disturbance. A gradual increase of HCC along the gradient of human disturbance was not observed in *M. murinus*. Moreover, the general body condition of grey mouse lemurs was best in the most disturbed habitat. Unlike the findings of previous studies (Raharivololona and Ganzhorn, 2009), the overall parasitism patterns observed among the four populations of *M. murinus* were comparable. Surprisingly, the three health indicators were comparable among the fat-tailed dwarf lemur populations exposed to different levels of anthropogenic activities. However, as reported for several other species, season, sex and age seem to play a role in influencing health indicators in both species (ground squirrels, Boswell *et al*., 1994; mammals, Schalk and Forbes, 1997; non-rodent mammals, Tilbrook *et al*., 2000).

The higher prevalence observed for males mouse lemurs during the dry season could be associated with a higher exposure to parasites because of their elevated activity (Eberle and Kappeler, 2004) and/or a higher susceptibility because of the immunosuppressive effect of high steroid hormone concentrations during this period (Perret, 1985). Although a seasonal comparison of the pattern of parasitism could not be achieved for *C. medius*, it is known for several species that the immune function declines during hibernation and can therefore increase individual susceptibility but also the virulence of pathogens (reviewed by Martinez-Bakker and Helm, 2015). However, at the population level, the effect of a reduced immunity could be compensated by a reduced exposure of hibernators to parasite infections. Moreover, the constantly higher prevalence seen in *M. murinus* in comparison to *C. medius* could be attributed to the fact that while *C. medius* hibernates, mouse lemurs remain partly active during the cold dry season and thus have a higher probability of encountering parasites throughout the year.

Invariant levels of the health indicators between sites may primarily result from selective disappearance (Romero, 2004). It is possible that high selective pressure could have caused death or emigration of the more sensitive individuals. In a previous study of the N5 and CS7 populations, Hämäläinen *et al*. (2014) confirmed that a relatively high body condition was required for *M murinus* to reach an old age in the wild. Anthropogenic disturbance might affect the required threshold level for survival, and this might explain the better body condition of *M. murinus* in KV. Several other factors that could explain the observed results, such as dietary adaptation, interspecific competition or the social context, are discussed below.

### Dietary adaptation

Dietary adaptation might explain the lack of differences in health indicators observed among the study populations, as well as the better body condition of KV mouse lemurs. When faced with a decrease in food availability, animals can alter their feeding behaviour to meet energetic demands either by broadening their dietary spectrum or by exploiting a subset of their original diet more extensively (Onderdonk and Chapman, 2000; Nakagawa *et al*., 2007; Gibson, 2011). Animals that are not able to adjust their diet may experience a rapid decline in population size (Chapman *et al*., 2006). At the physiological level, one potential explanation for this outcome is the synergistic effect of parasitism and dietary stress on hosts.

While the immunosuppressive effect of prolonged food shortage will increase parasitism, high parasite loads could consecutively increase energy demands on the host and aggravate the effect of food scarcity. Changes in nutritional habits have been widely noted in wild populations in changing conditions. For instance, diademed sifakas (*Propithecus diadema*) consume a greater amount of mistletoe (*Bakerella*) in fragmented areas compared with continuous forest to compensate for the low availability of suitable fruiting tree species in fragments (Irwin, 2008b). Cheirogaleids were also shown to undergo dietary shifts in fragmented habitats; mouse lemurs increased their arthropod consumption, and dwarf lemurs (*Cheirogaleus sibreei* and *Cheirogaleus crossleyi*) shifted from a frugivorous to a more omnivorous diet in fragmented forest (Crowley *et al*., 2013). Therefore, the abundance of arthropods near the forest edge was proposed to stabilize population density in edge habitats (Lehman *et al*., 2006).

The intraspecific variation of food tree density among sites might have facilitated a shift in diet. Fat-tailed dwarf lemurs were observed to feed extensively on *Phyllanthus casticum* pulp at the end of the rainy season in KV and SV, while this tree species is found at a low density in N5 and is absent in CS7. Additionally, a lower density in disturbed sites might further facilitate the access to resources by the remaining individuals. However, we acknowledge the fact that the inventory of tree species eaten by *M. murinus* and *C. medius* (Fietz and Ganzhorn, 1999; Dammhahn and Kappeler, 2008) was established in a continuous forest. Therefore, tree species that are potentially suitable for consumption in fragments and disturbed areas might be missing from this list. Thus, a detailed description of the feeding behaviour of both species across habitats with different levels of disturbance will be needed in further studies.

### Interspecific competition

Interspecific interactions, such as competition for resources or predation, can be perceived as non-negligible stressors and can trigger a similar effect to human disturbance for wildlife populations (Frid and Dill, 2002). Among the members of the cheirogaleid family, *C. medius* was observed partly to displace *M. murinus* on a local scale, and the latter was found to avoid direct competition with the closely related *Microcebus berthae* by spatial separation in an undisturbed habitat (Schwab and Ganzhorn, 2004). Schäffler *et al*. (2015) emphasized that interspecific interactions within the cheirogaleid family played an important role in shaping their community composition and that the relative abundance of each species depended on the degree of environmental disturbance. They demonstrated that despite an overlap in their diet, *M. murinus* and *M. berthae* could coexist in intact habitat because of predation by *Mirza coquereli* on *M. murinus*. Therefore, *M. berthae* benefit from the spatial avoidance of predation pressure by avoiding competition. In return, *M. murinus* avoid competition and intraguild predation by occupying habitats with high levels of human disturbance. Consequently, the physiological stress caused by human activities in disturbed areas might be balanced with the high pressure set by interspecific competition in more suitable habitats. In order to test this hypothesis, a study of the overall predation pressure between sites and a quantification and differentiation of human-induced stressors seems indicated.

### Social factors

Ranging patterns and population density are also known to affect parasitism patterns (Hudson *et al*., 2002; Nunn *et al*., 2003). Parasite prevalence, diversity and infection rates are correlated positively with host density and home range size (Morand and Poulin, 1998; Packer *et al*., 1999). The variation in capture success of mouse lemurs and dwarf lemurs among sites might indicate a constantly higher density of *M. murinus* in comparison to *C. medius* and decreasing population density with increased habitat disturbance in both species. This conclusion is concordant with previous findings of Schäffler *et al*. (2015) in the central Menabe region. However, the patterns of parasitism observed in *M. murinus* and *C. medius* do not seem to be associated with density-dependent factors, as our data indicated no association between parasitism and host density. For both species, the importance of density in parasite spread could be trumped by social factors that could influence patterns of parasite transmission by direct contact between individuals. *Microcebus murinus* have a promiscuous mating system, and females are known regularly to share sleeping sites (Radespiel, 2000). Despite the fact that they live in permanent pairs, an increase in contact rates between individuals of the fat-tailed dwarf lemur was observed during the mating season, when a high rate of extra-pair copulations was recorded as well (Fietz *et al*., 2000). The importance of social contacts in parasite transmission was highlighted by several studies (reviewed by Kappeler *et al*., 2015) for both directly and environmentally transmitted parasites (Drewe, 2010; MacIntosh *et al*., 2012; Rimbach *et al*., 2015).

A major limitation of our study is the relatively low and unbalanced sample size collected per site. Nonetheless, the limited capture success resulting in the restricted samples size gathered in the disturbed sites could be an indicator of the indirect long-term negative consequences of human activities and might imply that population sizes are lower where habitats are suboptimal (Schäffler *et al*., 2015). The significance of human presence might have a limited detrimental impact on the health of the study species, because direct encounter rates with humans are probably low for those small nocturnal animals which also do not suffer from direct hunting. Moreover, the forest concession is a reasonably continuous habitat, and no small fragment was listed among our study sites. However, the substantial differences in habitat structure observed among sites are at least partly attributable to human use of the areas. Our results thus suggest that even moderate habitat alteration might influence the population viability of flexible species.

### Conclusions

This study demonstrates that moderate human disturbance may have negligible influence on the general health status of species that are capable of adjusting to suboptimal conditions by behavioural or dietary flexibility. However, the relatively lower density of these species in disturbed environments indicates that human activities may negatively affect the long-term population viability of even resilient species. These findings highlight the need to limit human activities in natural areas and prioritize continuous pristine forests in conservation actions. Although health parameters are often easier to measure than population density or population decline, the present study emphasizes the degree of uncertainty associated with such shortcuts. Although an assessment of the general health of wild populations can be informative concerning their potential sensitivity to environmental change, our results indicate that basing conservation decisions solely on health information may overestimate the resilience of the population, thus increasing the risk of misinformed conservation decisions. We thus propose that, for the purposes of political decision-making processes, information on health parameters should be coupled with a study of their fitness consequences as well as other indicators of population viability.

## Summary

We examined the effect of anthropogenic disturbance on stress, body condition and parasitism in mouse lemurs and fat-tailed dwarf lemurs. The results revealed no effect of human disturbance in both species. Yet, a difference in relative density among sites suggests that extended habitat deterioration could hamper long-term viability of populations.

## Supplementary material

Supplementary material is available at *Conservation Physiology* online.

## Funding

This work was supported by the Deutscher Akademischer Austausch Dienst (A/12/90426) and the Deutsche Forschungsgemeinschaft (KR 3834/4-1).

## Supplementary Material

Supplementary Data
