# Supplementary material for: Does habitat disturbance affect stress, body condition and parasitism in two sympatric lemurs?
Source: Conserv Physiol. 2016 Sep 10;4(1):cow034. doi: 10.1093/conphys/cow034 (PMC5020880; doi:10.1093/conphys/cow034)
Supplement: Supplementary Data [file cow034_supplementary_material.docx]

**Supplementary material**

**Table S1** List and estimated density of trees consumed by *C. medius* and *M. murinus* in Kirindy C.N.F.E.R.E.F. The list was retrieved from Fietz and Ganzhorn (1999) and Dammhahn and Kappeler (2008). An update of classification and scientific nomenclature of the trees species was obtained from a phonological study conducted in Kirindy forest in 2013.

|  |  |  | Density (Trees/ha) | | | |
| --- | --- | --- | --- | --- | --- | --- |
| Species | Family | Local name | N5 | CS7 | SV | KV |
| *Terminalia boivinii* | Combretaceae | Amaninomby | 258 | 745 | 133 | 355 |
| *Strychnos henningsii* | Loganiaceae | Tsivoanino | 484 | 395 | 840 | 59 |
| *Strychnos decussata* | Loganiaceae | Hazomby | 1493 | 4680 | 3244 | 131 |
| *Rothmannia sp* | Rubiaceae | Manamakanamy | 159 | 0 | 4 | 0 |
| *Phylloctenium decaryanum* | Bignoniaceae | Pitikala | 250 | 50 | 62 | 10 |
| *Phyllarthron subumbellatum* | Bignoniaceae | Tohiravy | 49 | 140 | 27 | 6 |
| *Phyllanthus casticum* | Euphorbiaceae | Sanira petite feuille | 14 | 0 | 178 | 92 |
| *Peponidium* sp | Rubiaceae | Kitatamalando | 49 | 20 | 49 | 7 |
| *Ochna pervilleana* | Ochnaceae | Boramena | 882 | 2705 | 71 | 120 |
| *Noronhia sp* | Oleaceae | Tsilaitsy | 21 | 105 | 58 | 0 |
| *Memecylon* sp | Melastomataceae | Tsivoany | 284 | 45 | 1431 | 29 |
| *Macphersonia gracilis* | Sapindaceae | Tsingena | 2927 | 15 | 778 | 3 |
| *Hyperacanthus* sp | Rubiaceae | Piripitsokala | 484 | 225 | 129 | 17 |
| *Hymenodictyon occidentale* | Rubiaceae | Beholitsy | 205 | 390 | 80 | 37 |
| *Hilsenbergia capuronii* | Boraginaceae | Hazomboenga | 27 | 320 | 4 | 38 |
| *Grewia* sp | Malvaceae | Sele nala | 170 | 0 | 0 | 3 |
| *Grewia* sp | Malvaceae | Sele sele | 63 | 110 | 31 | 93 |
| *Grewia lavanalensis* | Malvaceae | Sele be | 23 | 30 | 289 | 37 |
| *Grewia cyclea* | Malvaceae | Latabarika | 210 | 270 | 31 | 41 |
| *Grewia aff. voloina* | Malvaceae | Sele pitiky | 286 | 285 | 329 | 116 |
| *Euonymus elaeodendroides* | Celestraceae | Maronono | 552 | 105 | 13 | 7 |
| *Diospyros tropophylla* | Ebenaceae | Maintifototsy | 355 | 250 | 111 | 80 |
| *Commiphora aprevalii* | Burseraceae | Sarisakoambanditsy | 40 | 245 | 89 | 59 |
| *Clerodendrum aff. involucratum* | Verbenaceae | Ripiky | 48 | 180 | 58 | 24 |
| *Carphalea kirondron* | Rubiaceae | Menavony | 0 | 205 | 13 | 39 |
| *Capuronia benoistii* | Lythraceae | Mamiaho | 598 | 315 | 31 | 54 |
| *Canthium sp* | Rubiaceae | Fatekahitsy | 120 | 485 | 324 | 18 |
| *Brachylaena sp* | Asteraceae | Vahirano | 653 | 375 | 71 | 49 |
| ? | ? | Belohaliky | 13 | 95 | 13 | 33 |
| *?* | ? | Hazonkondoky | 529 | 275 | 222 | 1 |
| *?* | ? | Manoro | 18 | 0 | 0 | 0 |
| *?* | ? | Vahipindy mena | 594 | 0 | 0 | 1 |

**Table S2** Number of distinct individuals of different sex-age classes captured at each site (AM: Adult male; AF: Adult female; JM: Juvenile male; JF: Juvenile female) along with the number of samples for HCC (hair samples), SMI (morphometric and body mass measurements) and parasitism (fecal samples) assessment.

| Numbers of individuals (hair samples/morphometric measurements/feacal samples) | | | | | | | | |
| --- | --- | --- | --- | --- | --- | --- | --- | --- |
| *Microcebus murinus* | | | | | *Cheirogaleus medius* | | | |
|  | N5 | CS7 | SV | KV | N5 | CS7 | SV | KV |
| **AM** | 104 (55/139/203) | 38 (29/40/68) | 35 (32/38/46) | 07 (07/07/09) | 41 (52/46/52) | 03 (02/02/03) | 05 (01/02/07) | 06 (07/06/06) |
| **AF** | 127 (67/175/265) | 35 (40/49/81) | 31 (31/35/47) | 11 (11/10/10) | 45 (53/46/65) | 09 (07/05/09) | 06 (06/06/04) | 07 (06/07/05) |
| **JM** | 131 (75/139/193) | 37 (38/50/33) | 29 (21/24/33) | 09 (09/09/05) | 12 (10/08/07) | 02 (03/02/02) | - | 01 (01/00/00) |
| **JF** | 87 (39/81/125) | 21 (18/20/20) | 23 (20/19/22) | 10 (10/10/07) | 29 (28/27/21) | 05 (04/05/02) | 03 (03/03/02) | 01 (01/01/01) |

**Table S3** Number of distinct individuals of each sex per season captured at each site (DM: Male for the dry season; DF: Female for the dry season; RM: Male for the rainy season; RF: Female for the rainy season) along with the number of samples used for HCC, SMI and patterns of parasitism assessment.

| Numbers of individuals (hair samples/morphometric measurements/feacal samples) | | | | | | | | |
| --- | --- | --- | --- | --- | --- | --- | --- | --- |
| *Microcebus murinus* | | | | | *Cheirogaleus medius* | | | |
|  | N5 | CS7 | SV | KV | N5 | CS7 | SV | KV |
| **DM** | 136 (72/168/300) | 49 (32/54/74) | 46 (40/49/67) | 08 (09/09/10) | 44 (57/50/54) | 04 (04/03/04) | 05 (01/02/07) | 07 (08/06/06) |
| **DF** | 163 (55/138/275) | 37 (24/34/64) | 39 (39/43/60) | 11 (12/12/11) | 52 (57/48/66) | 09 (08/07/09) | 08 (08/08/05) | 07 (06/07/05) |
| **RM** | 104 (58/110/97) | 33 (35/36/27) | 13 (13/13/11) | 07 (07/07/04) | 06 (05/04/05) | 01 (01/01/01) | - | - |
| **RF** | 103 (51/118/114) | 31 (34/35/37) | 12 (12/11/10) | 09 (09/08/06) | 25 (24/25/20) | 03 (03/03/02) | 01 (01/01/01) | 01 (01/01/01) |

**Table S4** Parameter estimates from full linear mixed models assessing variations of log [Hair cortisol concentration (HCC)] and log [general body condition (calculated as scaled mass index)] in *M. murinus* and *C. medius.*

|  | *Microcebus murinus* | | | | *Cheirogaleus medius* | | | |
| --- | --- | --- | --- | --- | --- | --- | --- | --- |
|  | Estimate | SE | *t* | *p* | Estimate | SE | *t* | *p* |
| **Hair cortisol** |  |  |  |  |  |  |  |  |
|  |  |  |  |  |  |  |  |  |
| Intercept | 2.203 | 0.130 | 16.998 | **<0.001** | 1.808 | 0.187 | 9.680 | **<0.001** |
| Site (ref. N5) CS7  SV  KV | 0.248  -0.026  0.231 | 0.113  0.123  0.181 | 2.187  -0.212  1.276 | **0.003**  0.832  0.203 | 0.348  0.157  -0.356 | 0.268  0.330  0.273 | 1.298  0.475  -1.306 | 0.196  0.635  0.193 |
| Season (ref. dry) | 0.245 | 0.139 | 1.758 | 0.079 | 1.366 | 0.217 | 6.288 | **<0.001** |
| Sex (ref. female) | 0.419 | 0.154 | 2.712 | **0.006** | 0.025 | 0.325 | 0.078 | 0.938 |
| Age (ref. juvenile) | -0.517 | 0.141 | -3.654 | **<0.001** | -0.436 | 0.204 | -2.139 | **0.038** |
| Sex*Season | -0.532 | 0.187 | -2.842 | **0.005** | -0.248 | 0.474 | -0.524 | 0.601 |
| Sex*Age | -0.191 | 0.190 | -1.005 | 0.315 | 0.297 | 0.360 | 0.824 | 0.401 |
|  |  |  |  |  |  |  |  |  |
| **Body condition** |  |  |  |  |  |  |  |  |
|  |  |  |  |  |  |  |  |  |
| Intercept | 4.041 | 0.015 | 268.058 | **<0.001** | 4.829 | 0.030 | 158.170 | **<0.001** |
| Site (ref. N5) CS7  SV  KV | 0.006  -0.022  0.131 | 0.014  0.016  0.027 | 0.447  -1.368  4.807 | 0.728  0.230  **<0.001** | -0.088  -0.009  -0.014 | 0.046  0.051  0.046 | -1.916  -0.180  -0.312 | 0.057  0.857  0.756 |
| Season (ref. dry) | 0.091 | 0.016 | 5.704 | **<0.001** | 0.206 | 0.035 | 5.818 | **<0.001** |
| Sex (ref. female) | 0.056 | 0.018 | 3.005 | **0.002** | -0.001 | 0.055 | -0.019 | 0.985 |
| Age (ref. juvenile) | 0.051 | 0.017 | 3.032 | **0.002** | -0.001 | 0.033 | -0.055 | 0.956 |
| Sex*Season | -0.101 | 0.022 | -4.622 | **<0.001** | 0.106 | 0.083 | 1.283 | 0.201 |
| Sex*Age | -0.043 | 0.022 | -1.929 | 0.054 | -0.042 | 0.061 | -0.688 | 0.492 |

**Table S5** Parameter estimates from full generalised linear mixed models assessing variations of multiple-morphotype infection rate and parasite morphotype richness in *M. murinus* and *C. medius.*

|  | *Microcebus murinus* | | | | *Cheirogaleus medius* | | | |
| --- | --- | --- | --- | --- | --- | --- | --- | --- |
|  | Estimate | SE | *z* | *p* | Estimate | SE | *z* | *p* |
| **Multiple-morphotype infection** | |  |  |  |  |  |  |  |
|  |  |  |  |  |  |  |  |  |
| Intercept | -1.550 | 0.223 | -6.954 | **<0.001** | -0.476 | 0.521. | -0.914 | 0.361 |
| Site (ref. N5) CS7  SV  KV | 0.456  0.021  0.448 | 0.195  0.225  0.432 | 2.239  0.095  1.037 | **0.019**  0.924  0.297 | 0.052  -0.907  -0.575 | 0.713  0.811  0.824 | 0.074  -1.118  -0.698 | 0.941  0.264  0.485 |
| Season (ref. dry) | -0.172 | 0.229 | -0.751 | 0.452 | - | - | - | **-** |
| Sex (ref. female) | 0.900 | 0.263 | 3.425 | **<0.001** | -1.374 | 1.198 | -1.147 | 0.251 |
| Age (ref. juvenile) | 0.397 | 0.238 | 1.666 | 0.096 | -0.893 | 0.611 | -1.461 | 0.144 |
| Sample mass | 0.025 | 0.070 | 0.356 | 0.721 | -0.237 | 0.215 | -1.100 | 0.271 |
| Sex*Season | -0.901 | 0.335 | -2.689 | **0.007** | - | - | - | - |
| Sex*Age | -0.172 | 0.308 | -0.558 | 0.576 | 2.087 | 1.280 | 1.631 | 0.103 |
|  | |  |  |  |  |  |  |  |
| **Parasite morphotype richness** | |  |  |  |  |  |  |  |
|  | |  |  |  |  |  |  |  |
| Intercept | -0.269 | 0.097 | -2.272 | **0.005** | 0.048 | 0.280 | 0.173 | 0.863 |
| Site (ref. N5) CS7  SV  KV | 0.147  0.034  0.164 | 0.087  0.099  0.188 | 1.683  0.341  0.874 | 0.092  0.733  0.382 | -0.216  -0.413  -0.268 | 0.401  0.400  0.415 | -0.538  -1.031  -0.643 | 0.591  0.302  0.520 |
| Season (ref. dry) | -0.190 | 0.102 | -1.860 | 0.063 | - | - | - | **-** |
| Sex (ref. female) | 0.384 | 0.115 | 3.333 | **<0.001** | -0.534 | 0.535 | -0.999 | 0.317 |
| Age (ref. juvenile) | 0.217 | 0.105 | 2.056 | **0.040** | -0.513 | 0.325 | -1.578 | 0.115 |
| Sample mass | 0.015 | 0.029 | 0.508 | 0.611 | -0.081 | 0.111 | -0.723 | 0.469 |
| Sex*Season | -0.312 | 0.146 | -2.137 | **0.033** | **-** | - | - | - |
| Sex*Age | -0.057 | 0.134 | -0.423 | 0.672 | 1.138 | 0.588 | 1.933 | 0.053 |

**Table S6** Parameter estimates from full generalised linear mixed models assessing variations of parasite prevalence in *M. murinus* and *C. medius.*

|  | *Microcebus murinus* | | | | *Cheirogaleus medius* | | | |
| --- | --- | --- | --- | --- | --- | --- | --- | --- |
|  | Estimate | SE | *z* | *p* | Estimate | SE | *z* | *p* |
| *Overall prevalence* |  |  |  |  |  |  |  |  |
| Intercept | 0.106 | 0.184 | 0.576 | 0.564 | 0.458 | 0.532 | 0.860 | 0.390 |
| Site (ref. N5) CS7  SV  KV | 0.239  0.085  -0.069 | 0.197  0.219  0.422 | 1.212  0.391  -0.164 | 0.225  0.696  0.870 | -0.061  -0.429  -0.298 | 0.640  0.654  0.687 | -0.095  -0.656  -0.434 | 0.925  0.512  0.665 |
| Season (ref. dry) | -0.287 | 0.202 | -1.417 | 0.156 | - | - | - | - |
| Sex (ref. female) | 0.670 | 0.237 | 2.828 | **0.004** | 0.193 | 0.938 | 0.206 | 0.837 |
| Age (ref. juvenile) | 0.275 | 0.204 | 1.345 | 0.179 | -0.763 | 0.600 | -1.273 | 0.203 |
| Sample mass | 0.089 | 0.067 | 1.333 | 0.182 | -0.205 | 0.198 | -1.035 | 0.301 |
| Sex*Season | -0.648 | 0.296 | -2.193 | **0.028** | - | - | - | - |
| Sex*Age | 0.120 | 0.285 | 0.420 | 0.674 | 0.582 | 1.028 | 0.566 | 0.571 |
|  |  |  |  |  |  |  |  |  |
| *Hymenolepis* |  |  |  |  |  |  |  |  |
| Intercept | -0.738 | 0.180 | -4.088 | **<0.001** | -0.019 | 0.556 | -0.034 | 0.973 |
| Site (ref. N5) CS7  SV  KV | -0.063  -0.626  0.003 | 0.171  0.208  0.387 | -0.367  -3.015  -0.010 | 0.713  **0.002**  0.992 | -3.452  -1.044  -0.671 | 2.207  0.871  0.881 | -1.564  -1.199  -0.761 | 0.118  0.231  0.446 |
| Season (ref. dry) | -0.118 | 0.199 | -0.595 | 0.552 | - | - | - | **-** |
| Sex (ref. female) | 0.301 | 0.226 | 1.333 | 0.182 | -0.813 | 1.027 | -0.792 | 0.428 |
| Age (ref. juvenile) | 0.317 | 0.201 | 1.576 | 0.115 | -1.304 | 0.664 | -1.964 | **0.049** |
| Sample mass | 0.069 | 0.062 | 1.101 | 0.271 | -0.288 | 0.243 | -1.185 | 0.236 |
| Sex*Season | -0.383 | 0.289 | -1.323 | 0.186 | - | - | - | - |
| Sex*Age | 0.012 | 0.268 | 0.046 | 0.964 | 1.364 | 1.140 | 1.197 | 0.231 |
|  |  |  |  |  |  |  |  |  |
| *Subulura* |  |  |  |  |  |  |  |  |
| Intercept | -1.261 | 0.207 | -6.103 | **<0.001** | -0.703 | 0.564 | -1.245 | 0.213 |
| Site (ref. N5) CS7  SV  KV | 0.719  0.713  0.426 | 0.193  0.217  0.427 | 3.729  3.292  0.997 | **<0.001**  **<0.001**  0.319 | -0.052  0.035  0.020 | 0.768  0.727  0.792 | -0.067  0.048  0.026 | 0.946  0.961  0.980 |
| Season (ref. dry) | -0.113 | 0.221 | -0.514 | 0.607 | - | - | - | **-** |
| Sex (ref. female) | 0.914 | 0.250 | 3.658 | **<0.001** | 0.260 | 0.955 | 0.273 | 0.785 |
| Age (ref. juvenile) | 0.119 | 0.224 | 0.533 | 0.594 | -0.857 | 0.651 | -1.318 | 0.188 |
| Sample mass | 0.105 | 0.068 | 1.537 | 0.124 | -0.231 | 0.231 | -1.002 | 0.316 |
| Sex*Season | -1.156 | 0.327 | -3.537 | **<0.001** | - | - | - | - |
| Sex*Age | 0.0006 | 0.296 | 0.002 | 0.998 | 0.529 | 1.061 | 0.499 | 0.618 |

**Table S6** continued.

|  | *Microcebus murinus* | | | | *Cheirogaleus medius* | | | |
| --- | --- | --- | --- | --- | --- | --- | --- | --- |
|  | Estimate | SE | *z* | *p* | Estimate | SE | *z* | *p* |
| *Trichuris* |  |  |  |  |  |  |  |  |
| Intercept | -3.118 | 0.386 | -8.067 | **<0.001** | -8.018 | 1.752 | -4.575 | **<0.001** |
| Site (ref. N5) CS7  SV  KV | 0.008  -0.572  0.101 | 0.379  0.419  0.826 | 0.020  -1.364  0.123 | 0.984  0.172  0.902 | -2.296  0.169  -1.317 | 3.097  2.237  3.107 | -0.741  0.076  -0.424 | 0.459  0.940  0.672 |
| Season (ref. dry) | - | - | - | - | - | - | - | **-** |
| Sex (ref. female) | 1.002 | 0.297 | 3.373 | **<0.001** | -0.188 | 1.539 | -0.122 | 0.903 |
| Age (ref. juvenile) | 0.526 | 0.269 | 1.954 | 0.051 | -0.389 | 1.498 | -0.260 | 0.795 |
| Sample mass | 0.174 | 0.114 | 1.523 | 0.128 | 0.418 | 0.577 | 0.725 | 0.468 |
| Sex*Season | - | - | - | - | - | - | - | - |
| Sex*Age | - | - | - | - | - | - | - | - |
|  |  |  |  |  |  |  |  |  |
| *Ascaris* |  |  |  |  |  |  |  |  |
| Intercept | -3.862 | 0.528 | -7.320 | **<0.001** | -10.263 | 2.220 | -4.624 | **<0.001** |
| Site (ref. N5) CS7  SV  KV | 0.875  0.600  1.999 | 0.324  0.369  0.511 | 2.697  1.627  3.915 | **0.007**  0.103  **<0.001** | 0.315  -0.881  0.498 | 2.621  3.409  2.697 | 0.120  -0.258  0.185 | 0.904  0.796  0.854 |
| Season (ref. dry) | -1.702 | 0.747 | -2.279 | **0.023** | - | - | - | - |
| Sex (ref. female) | 0.802 | 0.590 | 1.361 | 0.173 | -1.151 | 1.841 | -0.082 | 0.935 |
| Age (ref. juvenile) | 0.891 | 0.560 | 1.591 | 0.111 | 0.443 | 1.950 | 0.227 | 0.820 |
| Sample mass | -0.236 | 0.127 | -1.855 | 0.063 | -0.283 | 0.896 | -0.315 | 0.753 |
| Sex*Season | 0.926 | 0.896 | 1.033 | 0.301 | - | - | - | - |
| Sex*Age | -0.667 | 0.661 | -1.009 | 0.313 | - | - | - | - |
